# MTMS-Based Aerogel Constructs for Immobilization of Plant Hairy Roots: Effects on Proliferation of *Rindera graeca* Biomass and Extracellular Secretion of Naphthoquinones

**DOI:** 10.3390/jfb12010019

**Published:** 2021-03-05

**Authors:** Bartosz Nowak, Mateusz Kawka, Kamil Wierzchowski, Katarzyna Sykłowska-Baranek, Maciej Pilarek

**Affiliations:** 1 Faculty of Chemical and Process Engineering, Warsaw University of Technology, Waryńskiego 1, 00-645 Warsaw, Poland; bartosz.nowak.dokt@pw.edu.pl (B.N.); kamil.wierzchowski.dokt@pw.edu.pl (K.W.); 2 Department of Pharmaceutical Biology and Medicinal Plant Biotechnology, Faculty of Pharmacy, Medical University of Warsaw, Banacha 1, 02-097 Warsaw, Poland; mateusz.kawka@wum.edu.pl (M.K.); katarzyna.syklowska-baranek@wum.edu.pl (K.S.-B.)

**Keywords:** MTMS aerogel, naphthoquinones, hairy (transgenic) roots, plant biomass immobilization, polyurethane foam (PUF), in situ extraction

## Abstract

Unique biosynthetic abilities revealed by plants determine in vitro cultures of hairy roots as a suitable source of pharmaceutically relevant bioactive compounds. The basic aim of the study was to examine the applicability of aerogel composed of methyltrimethoxysilane (MTMS) for immobilization of *Rindera graeca* hairy roots by identifying quantitative effects of biomass proliferation and naphthoquinones extracellular secretion in the aerogel-supported culture system. *R. graeca* hairy roots were simultaneously cultured for 28-days, as (i) nonimmobilized biomass (reference system), (ii) biomass immobilized on macroporous polyurethane foam (PUF), (iii) biomass with disintegrated MTMS aerogel, (iv) biomass immobilized on polypropylene (PP) fibers (as control), and (v) biomass immobilized on monolithic PP-reinforced MTMS aerogel. MTMS aerogel exhibited high level of biocompatibility toward *R. graeca* hairy roots which grew into the structure of monolithic aerogel-based constructs. Monolithic MTMS-based constructs significantly promoted the proliferation of hairy roots, resulting in 55% higher fresh mass than the reference system. The highest level of naphthoquinones productivity, i.e., 653 µg g_DW_^−1^, was noted for PUF-supported culture system.

## 1. Introduction

Plant secondary metabolites, produced with unique biosynthetic machinery of metabolic pathways, are a natural and renewable source of bioactive compounds for the biopharmaceutical industry [1]. Progress achieved in plant biotechnology has resulted in attractive alternatives to soil cultivation, as well as introducing novel in vitro bioengineering solutions for maximization of biologically active small-molecule compounds productivity [2]. The biotechnological approaches for plant secondary metabolites production are characterized by two main commercialization bottlenecks: (i) low yield of target compound biosynthesis and (ii) low purification yield [3]. Biomass immobilization and in situ product removal were demonstrated as the most distinguishing in terms of effectivity [4,5]. Both techniques, recognized well for their implementation in classic in vitro cultures of isolated plant cells, e.g., callus tissue cells, are so far less familiar in the field of plant organs bioprocessing, e.g., hairy (transgenic) roots.

Due to the latest progress in the comprehension of their molecular mechanism of action, naphthoquinone derivatives are currently becoming an increasingly fruitful source of novel drug candidates and, inevitably, the subject of emerging interest of pharmaceutical industry [6]. From the chemical point of view, naphthoquinones are shikonin derivatives, mainly, which are highly lipophilic pigments with wide profile of bioactivity [7,8,9].

Many previously published data concerning biomaterial screening explicitly tailored for application in various systems for in vitro culture of plant biomass. Nevertheless, the vast majority of literature reports presented only polyurethane or polypropylene foams/meshes as biocompatible materials intensifying in vitro production of secondary plant metabolites [10,11,12,13].

Our original idea is to examine a microporous organosilica-based aerogel as biomaterial suitable for simultaneous immobilization of hairy roots and in situ extraction of extracellularly secreted bioproducts. Due to the conceptual novelty of presented solution and limited literature in the field, we present organosilica-based aerogels as constructs positively characterized by their biocompatibility with plant tissue/organs, high porosity, highly developed specific surface area, as well as an affinity toward nonionic, lipophilic organic metabolites. In this context, organosilica-based aerogels with their hallmark wide range of surface modifications, allow to provide unique conjunction of such features and create an opportunity for directed design and adjustment of interactions between plant biomass, secondary metabolites and applied aerogel-based construct, observed within the aerogel-supported culture system.

The aim of the presented research was the feasibility study of a novel concept in producing plant-derived naphthoquinones with an application of organosilica-based aerogel constructs made of methyltrimethoxysilane (MTMS), as simultaneous support for *Rindera graeca* (Boraginaceae) hairy roots immobilization and in situ solid-phase extractant of extracellularly secreted naphthoquinones. The silica aerogel characterized by more defined functionality in plant roots in vitro cultures was compared with constructs made of consumer-grade polyurethane foam (PUF).

## 2. Materials and Methods

### 2.1. Materials Synthesis and Their Basic Characterization

Consumer-grade, resilient, macroporous PUF was purchased from a local reseller in cylindrical blocks.

Aerogel materials were synthesized via two-step acid-base sol-gel method. Trimethoxymethylsilane (MTMS, Sigma-Aldrich, St. Louis, MO, USA), as a precursor, and methanol (Stanlab), as a solvent, were used as received. Ammonia water (Eurochem BGD) and oxalic acid (Sigma-Aldrich), were applied as catalysts. First, MTMS was mixed with methanol and 0.01 M oxalic acid to initiate the hydrolysis reaction. After 60 min, the addition of the 1 M ammonium hydroxide significantly shifted the pH level, which began sol condensation. The following volume ratios were applied: MTMS:methanol:oxalic acid:ammonia equal to 1.0:2.0:0.8:0.8, what resulted in the following values of molar ratios for all the components, i.e., MTMS:methanol:water:oxalic acid:ammonia, as 1:7.05:12.53:0.01:0.114, respectively. Finally, for structure strengthening, the gelling solution was poured onto chemically-inert polypropylene (PP) fibers, to obtain fiber-reinforced MTMS-based aerogel. Prior to use, melt-blown PP fibers were soaked in 2-propanol to allow better access by reagents during gelation. Next, the condensed but wet MTMS-based gel was dried in ambient pressure at 50 °C for 24 h, and then at 100 °C to evaporate any residual liquids from pores of the aerogel. Mass ratio of PP fibers to MTMS aerogel was 1:5.

Basic characterization of materials covers SEM imaging (TM1000, Hitachi) and FT-IR (diamond crystal ATR FT-IR Nicolet iS20, Thermo Fisher Scientific, Waltham, MA, USA) for structural and chemical analysis, respectively. For SEM analysis, material samples were first sputtered with conductive gold nanolayer.

Apparent density and porosity were designated from at least five material samples by measuring their volume and mass. Porosity was calculated from the following equation:

*ε* = 1 − (*m* × *V*^−1^) × *ρ*_s_^−1^ [%] (1)

where *ε* is porosity, *m* is the sample mass, *V* is the sample volume, *ρ*_s_ is material skeletal density. Skeletal density was designated using a helium pycnometer and equalled 1.21 g cm^−3^ for PU, 1.20 g cm^−3^ for MTMS and 0.91 g cm^−3^ for PP.

Water in air contact angle was measured via a sessile drop method using OCA 25 goniometer (DataPhysics Instruments, Filderstadt, Germany). For each material, the contact angle was designated using 10 µL volume droplets, on five different spots and each droplet was measured three times to eliminate the influence of oscillation.

For the estimation of adsorption capacity of PUF and MTMS aerogel, as a mass of liquid per gram of material, for olive oil, rapeseed oil and water, five samples with known-weight were placed in tested liquid phases for 24 h. Adsorption capacity was calculated from the following equation:

adsorption capacity = (mass_wet material_ − mass_dry material_) × mass_dry material_^−1^ [g g^−1^] (2)

PUF, MTMS aerogel, PP fibers, and PP-reinforced MTMS aerogel were further individually investigated in *R. graeca* hairy roots culture.

### 2.2. Biomass and In Vitro Culture of Hairy Roots

Transgenic root line of *R. graeca*, originally established and developed via biotechnological methods as described by Sykłowska-Baranek et al. [14], was subjected to current experiments. Hairy roots were maintained in 250 cm^3^ Erlenmeyer flasks containing 50 cm^3^ of hormone-free DCR medium [15]. Culture flasks were agitated on an oscillatory shaker (INFORS AG) at 105 rpm and 24 °C, in darkness. Routine subculturing was performed every four weeks.

### 2.3. Experimental Procedures

Culture of nonimmobilized biomass was considered as the reference system.

In the case of material-supported cultures, presterilized PUF (cuboid sector, 3 × 3 × 0.5 cm, 0.2 g), MTMS aerogel (dried and powdered, 0.9 g), PP fibers (round sector, φ = 3 cm, 0.2 g) and PP-reinforced MTMS aerogel (round plate, φ = 3 cm, 0.5 cm of thickness, 0.9 g), were separately placed on the surface of 50 cm^3^ DCR culture medium inside 250 cm^3^ Erlenmeyer flasks to establish four independent culture systems. PP-fibers were tested only as solid-state reinforcement for mechanical stabilization of fragile MTMS aerogel, not as the material actively supporting hairy roots proliferation or in situ extraction of naphthoquinones. Therefore, culture supported only with PP fibers was considered as the control system, which identifies the possible negative effects on biomass, as well as positive influence on the 3-D structure of monolithic constructs of PP-reinforced MTMS aerogel.

The inoculum, 0.5 g of 28-days old *R. graeca* transgenic roots, was placed directly on the upper surface of the tested constructs. Next, the culture systems were continuously incubated for 28 days on oscillatory shaker (INFORS AG) at 105 rpm and 24 °C, in darkness.

After 28 days of culture, hairy roots were independently harvested from each culture system for fresh biomass weight analysis. Individually collected culture phases, i.e., 0.45 μm-filtered culture medium, as well as all materials, were individually separated and stored in −20 °C. Before extraction procedures, the hairy roots, as well as PUF, MTMS aerogel, PP fibers, PP-reinforced MTMS aerogel were lyophilized (Christ ALPHA 1-4 LSC). Lyophilized and micronized hairy root biomass, as well as not-treated culture medium, were extracted with *n*-hexane. Secondary metabolites accumulated in PUF, MTMS aerogel, PP fibers and PP-reinforced MTMS aerogel were independently extracted with HPLC-grade methanol. All extraction procedures were supported with sonication until the solvent color faded (Sonorex, Bandelin, Berlin, Germany).

### 2.4. Phytochemical Analysis of Extracts

Chromatographic procedure (RP-HPLC) and further analysis was carried out using DIONEX HPLC system connected with automated sample injector (ASI-100) and UVD 340S UV-Vis diode-array detector, under the following conditions: gradient elution—acetonitrile (60–80%)/0.04 M ortho-phosphoric acid (40–20%), flow rate 1.5 cm^3^ min^−1^, EC Nucleosil 120-7 ODS packed column (250 × 4.6 mm, 7 μm particles, 120 Å pores, Macherey-Nagel, Düren, Germany). Eluent absorbance was simultaneously monitored at 215 (as the reference wavelength), 237, 350, and 436 nm. Standards of shikonin derivatives of confirmed identity were used for peak identification according to the external standard methodology.

### 2.5. Mathematical Methods

The values of fresh biomass (FB_28d_) increase, which characterized the roots proliferation during a 28-day period of culture, were calculated from the following equation:

FB_28d_ = *m*_28d_ × *m*_0d_^−1^ [-] (3)

where *m*_28d_ is the fresh weight of transgenic roots at 28th day of culture, *m*_0d_ is the fresh weight of transgenic roots inoculum.

The yield of naphthoquinones production per unit mass (*Y_P/X_*), i.e., 1.0 g of dry biomass (*Y*_P/X_), were determined according to the following equation:

*Y*_P/X_ = *m*_n_ × (*DB*_28d_ − *DB*_0d_)^−1^ [g g_DW_^−1^] (4)

where *m*_n_ is the weight of naphthoquinones in the culture system, *DB*_28d_ is the dry weight of transgenic roots biomass measured at 28th day of culture, *DB*_0d_ is the dry weight of transgenic roots inoculum.

The values of specific growth rate (*μ*), which characterized the growth rate of hairy roots on applied constructs, were calculated from the following equation:

*μ* = (ln *m*_28d_ − ln *m*_0d_) × (_Δ_*t*)^−1^ [g g_DW_^−1^] (5)

where *t* is the time of culture.

## 3. Results

### 3.1. Material Characterization

**Figure 1:**
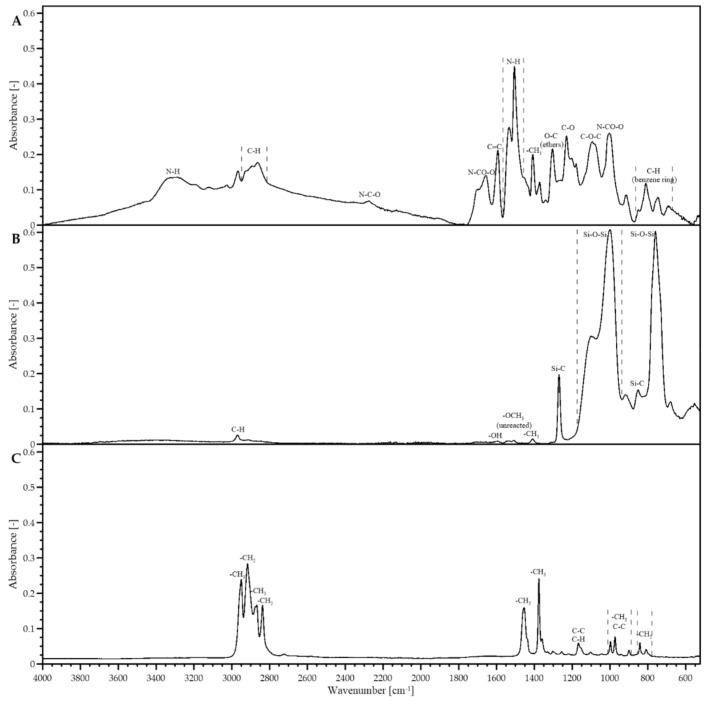
FT-IR spectra for studied materials: (**A**) polyurethane foam (PUF), (**B**) methyltrimethoxysilane (MTMS) aerogel and (**C**) polypropylene (PP) fibers.

Based on FT-IR spectra (Figure 1), functional group characteristics for all the basic materials, i.e., PUF, MTMS aerogel and PP fibers, were identified.

PUF (Figure 1A), as obtained in the polymerization of iso-cyanates and polyols, contained a repetitive urethane bond unit (i.e., -NHCOO-). The symmetric and asymmetric stretching vibrations of N-H correspond to broad peaks near 3322.9 and 1526.4 cm^−1^. Peaks at 1221.9 and 1726.3 cm^−1^ correspond to esters stretching vibration, i.e., C=O and C-O bonds. The peak at 916.8 cm^−1^ indicated the presence of a -NCOO- group. Rise at around 1604.4 cm^−1^ and broad region near 767–920 cm^−1^ region belonged to vibration of benzene ring. Polyurethane is visible in three peaks near 2979.5, 2929, and 2874 cm^−1^, corresponding to stretching vibrations of C-H, combining with -CH_3_ bending vibration noted at 1380 cm^−1^ [16,17]. Functional groups of PUF are responsible for its biocompatibility and wettability, with both organic and inorganic liquids.

Compared to PUF, FT-IR spectra of MTMS aerogel (Figure 1B) revealed its much simpler chemical composition. Peaks at 1123, 1030, and 753 cm^−1^ corresponded to Si-O-Si bonds characteristic for the silica structure. Hydrophobic -CH_3_ group was visible at 2970, 1274, and 853 cm^−1^ (-CH). Small peaks at around 1600 and 1410 cm^−1^ might indicate some unreacted silane groups or unhydrolyzed -OCH_3_ groups [18].

FT-IR spectra of PP fibers (Figure 1C) revealed visible peaks of -CH_3_ groups at 2952 and 2872 cm^−1^ and two for -CH_2_ at 2920 and 2839 cm^−1^. Peaks at 1456 and 1375 cm^−1^ were caused by -CH_3_ asymmetric and symmetric vibrations, respectively. A number of smaller peaks in the range of 800–1200 cm^−1^ correlated to other structural bonds of the polymer. The peak at 1160 cm^−1^ indicated C-C stretching, 998 cm^−1^ C-H bond or -CH_3_ group and 808 and 840 cm^−1^ -CH_2_ elements [19].

**Figure 2:**
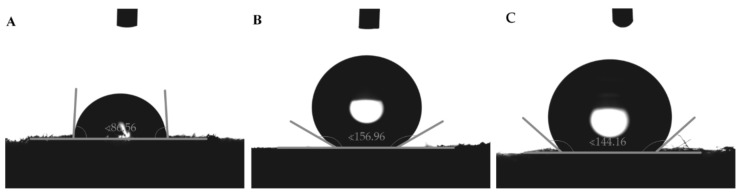
Goniometrical analysis of wettability performed for (**A**) PUF, (**B**) MTMS aerogel and (**C**) PP fibers.

Results of goniometrical analysis of wettability of PUF, MTMS aerogel and PP fibers are presented in Figure 2. In the case of PUF (Figure 2A), it is on the edge of hydro-phobicity/philicity with contact angle ca. 90°. Methyl groups present on the surface of MTMS aerogel lead to its high hydrophobicity, with water/air contact angle ca. 157° (Figure 2B). It is worth mentioning that the standard deviation of measurements performed for PUF indicates less regular surface structure, compared to MTMS aerogel. PP fibers (Figure 2C) also show a high contact angle of about 144° due to the presence of -CH_3_ groups and the fibrous structure which leads to a low wetted surface fraction. Similar to PUF, high standard deviation value was caused by chaotic structure of fibrous material.

Based on data reported in Table 1, all investigated materials may be recognized as light-weight constructs, reflecting low apparent density and high porosity. Although MTMS aerogels are commonly known as highly microporous structures, with porosity reaching 99.8%, the MTMS aerogel applied in the current study was characterized by 90.55% porosity. Significantly lower porosity value resulted from ambient pressure conditions of drying, which does not prevent volume shrinkage, but is recognized as more suited due to low cost. PUF was characterized by a very high porosity value, approximately 98.95%, which resulted from 10× lower apparent density than aerogel. PP fibers produced via melt-blown technique are loosely packed, which leads to low material density and high porosity equal to 97.27%. However, when used as structure enhancer, pores in between PP fibers are filled with MTMS aerogel. The 89.56% porosity is lower than the one obtained for MTMS aerogel and caused by fibers now occupying some of the aerogel pores.

**Figure 3:**
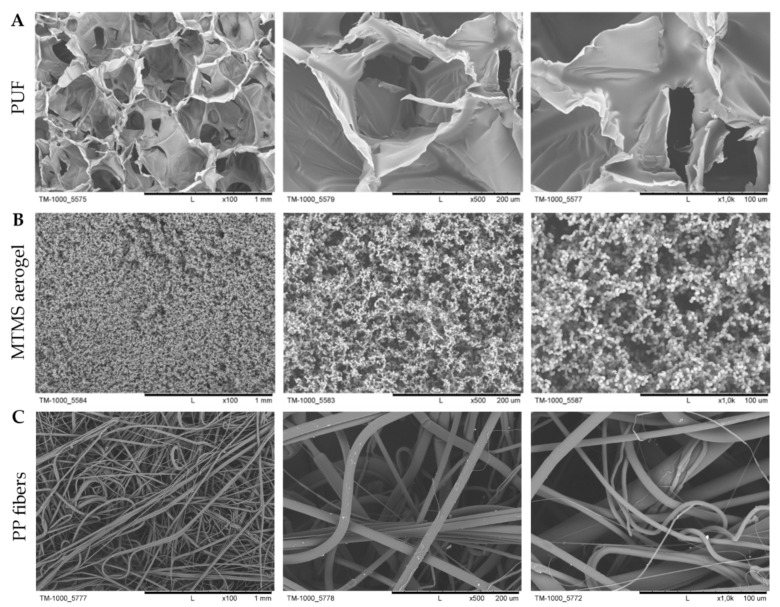
SEM micrographs of basic materials: (**A**) PUF, (**B**) MTMS aerogel and (**C**) PP fibers.

As shown in Figure 3, the difference in pore size between all materials was evident. In the case of PUF, the walls of macropores are flat and poreless, what clearly indicated on PUF porosity only on one scale. MTMS aerogel is microporous (Figure 3A), while PUF consists of macropores (Figure 3B). The structure of MTMS aerogel was fractal, built from secondary particles consisting of nano-scale primary particles of MTMS. Thus, the MTMS aerogel pore structure is also hierarchical leading to the high specific surface area. Chaotic structure of PP fibers (Figure 3C) is a characteristic one for materials obtained via melt-blown technique. The material is composed of entangled fibers of different diameter (i.e., mean fiber diameter equals approximately 5 µm). Pores between fibers create a space that can be filled with gel during condensation.

**Figure 4:**
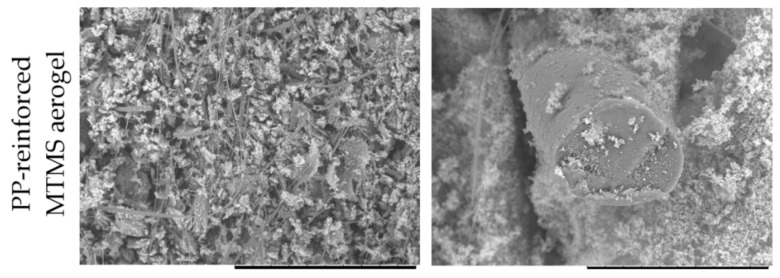
SEM micrographs of the cross-section of PP-reinforced MTMS aerogel.

Figure 4 shows SEM micrographs of PP-reinforced MTMS aerogel cross-section. Although the aerogel structure was shuttered during cutting, one can observe that organosilica is uniformly distributed in the fibrous material volume. Thanks to the precondensation soaking of the PP fibers in isopropanol, the reagents gain access to the fiber surface, which leads to better integrity with MTMS aerogel.

**Figure 5:**
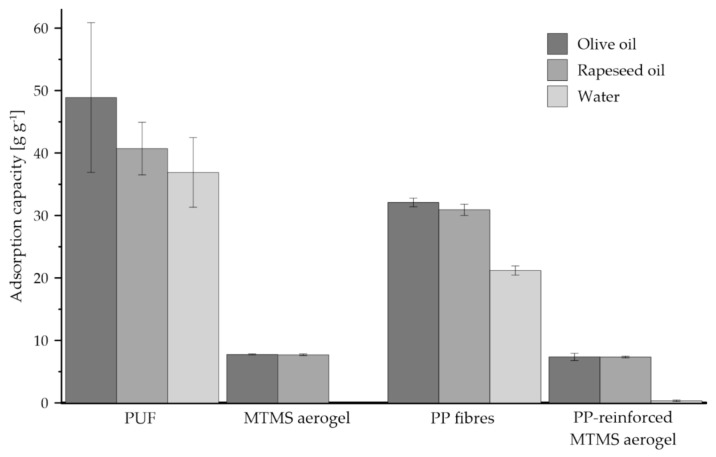
The values of adsorption capacity for PUF, MTMS aerogel, PP fibers and PP-reinforced MTMS aerogel exhibited toward olive and rapeseed oils, as well as water.

All investigated materials are open-porous, what directly corresponds with the results of adsorption capacity measurements presented in Figure 5. Macroporous PUF exhibited high adsorption capacity for both organic and inorganic liquids. Higher sorption was reached for oils, i.e., 50 and 41 g g^−1^ for olive and rapeseed oil, respectively. The adsorption capacity of 37 g g^−1^ determined for water indicates the more hydrophilic character of PUF. The microporous structure of MTMS aerogel leads to significantly lower value of sorption capacity, i.e., ca. 8 g g^−1^ for both tested oils, than corresponding values determined for PUF. Lack of water sorption clearly confirmed hydrophobicity of MTMS aerogel. Although PP fibers exhibit hydrophobic properties, the material does adsorb water, due to large pore size. Over 30 g g^−1^ adsorption of olive and rapeseed oil for PP fibers decrease to approximately 7.3 g g^−1^ while used in aerogel reinforcement. PP-reinforced MTMS aerogel shows not much lower oil adsorption capacity than native MTMS aerogel. As mentioned above (Table 1), it is caused by fibers occupying a fraction of aerogel pores. No significant adsorption of water (0.33 g g^−1^) makes it justified to treat PP fibers as the inert material only considering also their exiguous influence on aerogel oil adsorption capacity.

Determination of adsorption capacity for MTMS aerogel resulted in significantly lower level of error bars compared to PUF, what indicates more homogenous structure of MTMS aerogel than PUF. The differences in pore size noted for two compared materials (Figure 3) might affect adsorption capacity and soaking of already adsorbed liquid.

**Figure 6:**
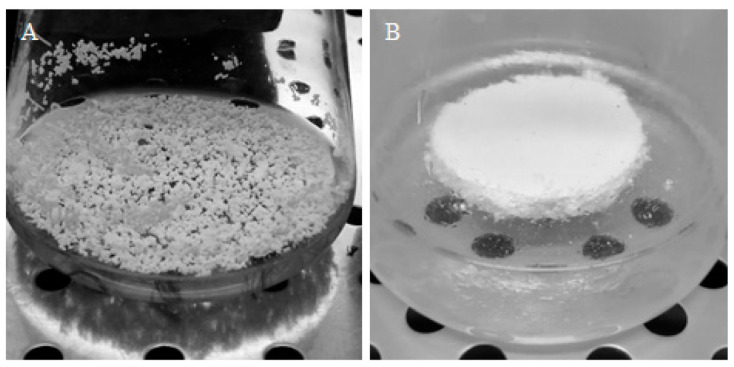
Two forms of MTMS aerogel-based materials applied into culture system: (**A**) disintegrated form and (**B**) monolithic PP-reinforced MTMS-based construct.

In the case of the present studies, the constructs made of PUF and MTMS aerogel were prepared as circular, monolithic plates/platforms, to offer a solid and flat surface which roots can inoculate, and further grow, with free access to both phases of air and aqueous culture medium (Figure 6A). Moreover, such forms of applied monolithic constructs should protect biomass from mechanical damage, as well as from negative effects of hydrodynamical shear stress caused by mixing. However, not every material tested in the current study remained monolithic during the whole culturing time. MTMS aerogel while applied as pure, i.e., nonreinforced with PP fibers, the material was fragile and easily disintegrated into small, i.e., 3–5 mm diameter, elements freely floating on the surface of culture medium (Figure 6B). The disintegration was caused by even mild agitation.

### 3.2. Cultures of R. graeca Hairy Roots on Applied Constructs

**Figure 7:**
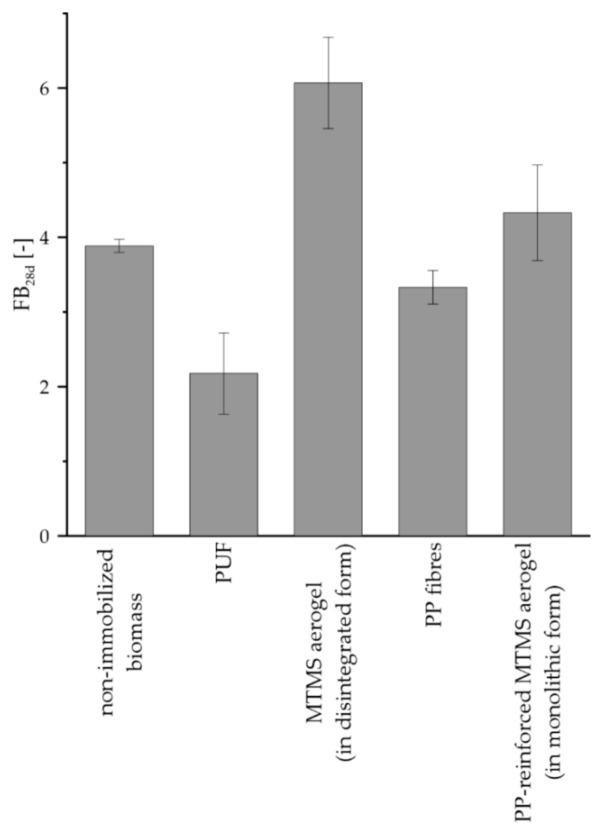
Comparison of the FB_28d_ values noted for *Rindera graeca* hairy roots proliferated in culture systems supported with tested materials.

The fresh biomass increase values noted for *R. graeca* hairy roots cultured on all types of applied constructs are shown in Figure 7. The highest value of FB_28d_ was noted in cultures performed with MTMS aerogel applied in disintegrated form (i.e., FB_28d_ = 6.07) and the value was over 55% higher than those determined for the reference, i.e., nonimmobilized culture (i.e., FB_28d_ = 3.88). Influence of PP fibers introduced into the culture on proliferation of hairy roots was negligible. However, the FB_28d_ value noted for culture system supported with PP-reinforced MTMS aerogel (i.e., FB_28d_ = 4.33) was significantly higher than the value noted for the reference culture. The lowest value of FB_28d_ was observed for cultures performed on PUF (i.e., FB_28d_ = 2.18), and such value was over 44% lower compared to the value of FB_28d_ noted for reference culture.

**Figure 8:**
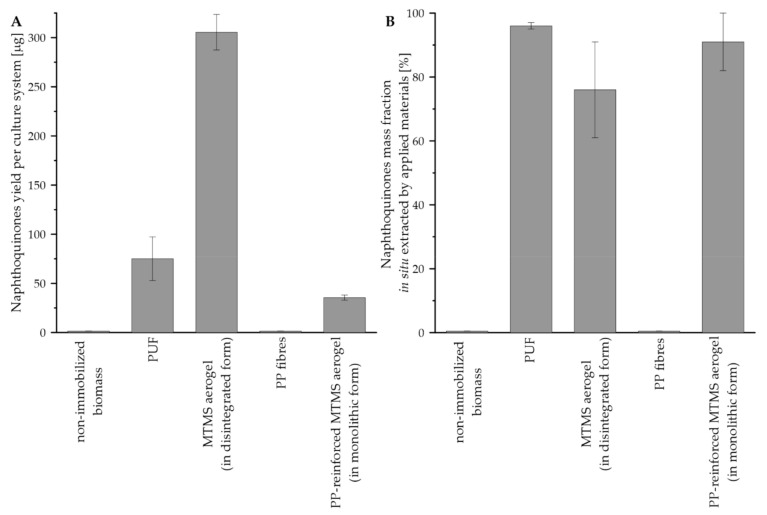
Values of the yield of naphthoquinones extracellularly secreted from *R. graeca* hairy roots: (**A**) naphthoquinones yield per culture system, (**B**) naphthoquinones mass fraction in situ extracted by materials supporting culture systems.

The values of naphthoquinones yield per culture system and the naphthoquinones mass fraction adsorbed on materials is presented in Figure 8. In the case of the reference, i.e., nonimmobilized, culture and the control culture performed on PP fibers, the level of naphthoquinones remained under the detection threshold. The highest value of naphthoquinones yield per culture system was noted for culture system supported with disintegrated MTMS aerogel (i.e., 305.4 µg), and such value was over four times higher than noted for system supported with PUF (i.e., 75.1 µg), as well as over eight times higher than the level reported for the system with PP-reinforced MTMS aerogel (i.e., 35.5 µg) (Figure 8A). In the case of naphthoquinones, the mass fraction in situ extracted by applied materials, the highest value of such parameter was detected for PUF-supported culture (i.e., 96%). The lowest value of the mass fraction was observed for culture systems supported with MTMS aerogel applied in disintegrated form (i.e., 76%) (Figure 8B).

Values of *Y*_P/X_ characterizing the productivity of naphthoquinones and values of *μ* characterizing proliferation of hairy roots in culture systems supported with tested materials are compared in Table 2. In the case of both, the reference system without biomass immobilization, as well as the control system containing PP fibers, values of *Y*_P/X_ equaled to 0 µg g_DW_^−1^, according to the lack of naphthoquinones detection. The highest value of *Y*_P/X_ was noted for hairy roots cultured on PUF (i.e., *Y*_P/X_ = 653.5 µg g_DW_^−1^). However, only a slightly lower value of *Y*_P/X_ was observed in the culture system supported by disintegrated MTMS aerogel (i.e., *Y*_P/X_ = 636.2 µg g_DW_^−1^). The level of *Y*_P/X_ noted for PP-reinforced MTMS aerogel (i.e., *Y*_P/X_ = 46.04 µg g_DW_^−1^) was significantly lower than data noted for PUF-supported system.

In the case of *μ* values, the reference culture system was characterized by *μ* equal to 2.019 × 10^−3^ h^−1^, and the control culture supported with PP fibers by *μ* equal to 1.790 × 10^−3^ h^−1^. The highest value was observed for hairy roots proliferated in the presence of disintegrated MTMS aerogel (i.e., *μ* = 2.678 × 10^−3^ h^−1^). Reinforcement of MTMS aerogel with PP fibers resulted in relatively low limitation of biomass proliferation (i.e., *μ* = 2.169 × 10^−3^ h^−1^). The lowest value of *μ*, i.e., 1.134 × 10^−3^ h^−1^, was noticed for the system supported with PUF.

## 4. Discussion

Organosilica aerogels are commonly recognized as highly porous, low dense, microporous materials, with a well-developed specific surface area. Up to now, organosilica aerogels have been applied as chemical and energy absorbers, catalyst carriers, filtration materials, building insulation, imaging devices, and even as drug-carriers in medicine delivery systems [20,21,22,23]. However, the application of organosilica aerogels as constructs for immobilization of plant hairy roots has not been studied so far. Moreover, effects of aerogel-based constructs exhibited on proliferation of transgenic roots or another type of in vitro cultured plant biomass, as well as their effects on the extracellular secretion of plant-derived metabolites, have not been identified, discussed nor analyzed up to date. Therefore, we compared our results with a range of other material-based constructs previously presented in literature focused on in vitro hairy root biomass bioengineering (Table 3).

In the case of cultures of *R. graeca* transgenic roots performed on PUF and MTMS aerogel, the most robust productivity of naphthoquinones was characterized by very close values of *Y*_P/X_ equal to ca. 650 µg g_DW_^−1^. In previously published reports, the highest value of *Y*_P/X,_ i.e., 11 976 µg g_DW_^−1^, has been reported for resveratrol production by *Arachis hypogaea* transgenic roots maintained on DIAION HP-20 polystyrene resin in 0.05 dm^3^ of MSV medium [24]. In the case of hairy roots immobilized on PUF, the highest values of *Y*_P/X_, i.e., 6400 µg g_DW_^−1^, has been reported by Srivastava et al. [11] for production of azadirachtin by hairy roots of *Azadirachta indica* performed in 1 dm^3^ of the classical MS medium. Thakore et al. [12] reported the value of *Y*_P/X_ equal to 1130 µg g_DW_^−1^ for ajmalicine production obtained in the system of *Catharanthus roseus* transgenic roots cultured on PUF in 3 dm^3^ of Gamborg B5/2 medium. In the case of other types of materials which have been applied for supporting bioprocessed hairy roots, the lower values of *Y*_P/X,_ i.e., 550 µg g_DW_^−1^, have been observed for diterpenoids production by *Salvia miltiorrhiza* transgenic roots immobilized on X-5 polystyrene and Amberlite XAD-4 resins performed in 0.05 dm^3^ of MS medium [25].

In the present study, for culture system supported with MTMS aerogel, *µ* equal to 2.68 × 10^−3^ h^−1^ was obtained. It was the second-highest value of *µ*, if compared to previously published data presented in Table 3. The only higher value of *µ*, i.e., 3.57 × 10^−3^ h^−1^, was reported for *Plumbago rosea* transgenic roots maintained on PP disk cultured in 1 dm^3^ of classic MS medium [13]. In the case of *µ* values observed for cultures of transgenic roots performed on PUF, the highest *µ*, i.e., 2.42 × 10^−3^ h^−1^, was reported by Srivastava et al. (2012) [11] for azadirachtin biosynthesis by *Azadirachta indica* transgenic roots in 1 dm^3^ of MS medium.

In the case of biomass proliferation, the system supported with PUF reached a 56% value obtained for the reference system. For PP-reinforced MTMS aerogel, 12% higher biomass growth than for the reference culture was detected. Remarkably, the highest hairy roots proliferation resulting in over 150% level related to the reference culture was noted for the system supported with MTMS-aerogel supporting culture in disintegrated form of 3–5 mm elements.

Among all constructs supporting cultures of *R. graeca* hairy roots, the most intensive proliferation of biomass was observed for the system supported with disintegrated MTMS aerogel (Table 2), and it resulted in ca. 150% higher value of biomass increase. Whilst, over 40% reduction in biomass increase in cultures supported with PUF was observed. In the case of the control culture, the PP fibers exhibited rather small effects on biomass proliferation, what resulted in only 15% reduction of biomass increase, if related to the system with PP-reinforced MTMS aerogel.

**Figure 9:**
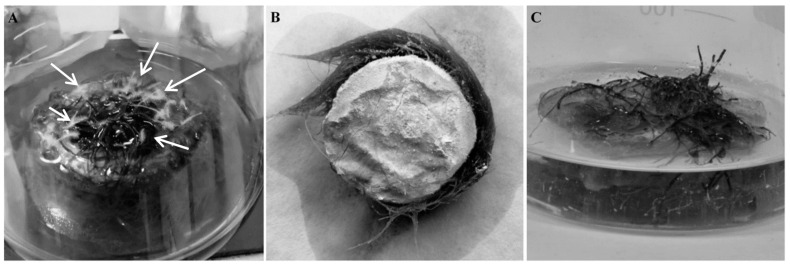
Morphology of *Rindera graeca* hairy roots proliferated on tested constructs made of monolithic PP-reinforced MTMS aerogel: (**A**) view from above with newly formed root hairs marked with white arrows, (**B**) bottom view; and PUF: (**C**) view from above.

While placed on the monolithic materials, hairy roots spread and covered only the top surface of the construct, until reaching its edge. Then, the roots elongated into aqueous culture medium (Figure 9A,B). It means that biomass was mechanically protected as long as it grew only on the surface of construct. Longer roots were exposed to negative effects that originated from hydrodynamics and mechanical agitation of the culture system. Finally, this resulted in hairy root collisions with the culture vessel walls leading to mechanical destruction of long hairy roots.

Visual analysis of the morphology of hairy roots cultured on the monolithic constructs revealed significant differences which hypothetically resulted from the type of applied material. *R. graeca* hairy roots grown on the PP-reinforced MTMS aerogel intensively developed aggregates of newly developed hairy roots with root hairs exactly in the regions where biomass was exposed to air, what can be easily seen as white-colored “hyphae-like” regions in Figure 9A. However, intensive growth of hairy roots with newly organized root hairs, was not observed for biomass cultured on PUF-based constructs (Figure 9C). Limitation in roots elongation was probably related to reduced availability of culture medium.

Based on data presented in Figure 7, it can be concluded that naphthoquinones were accumulated in all tested constructs, i.e., PUF, MTMS aerogel and PP-reinforced MTMS aerogel. Such effects resulted from high sorption capacity of PUF and MTMS exhibited toward organic compounds (Figure 5). PUF and monolithic MTMS constructs adsorbed over 90% of total naphthoquinones produced in the respective culture systems. MTMS aerogel applied in disintegrated form adsorbed only 76% of naphthoquinones fraction. Higher standard deviation values noted for both systems supported with MTMS aerogel, probably resulted from limited extraction of naphthoquinones from nanopores of MTMS aerogel (Figure 3).

**Figure 10:**
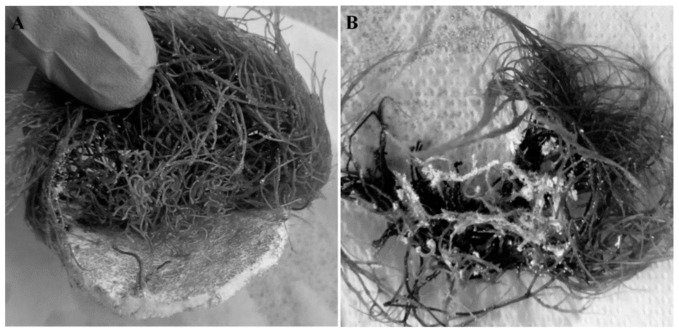
Biomass and biomaterial interaction observed for *R. graeca* hairy roots proliferated on PP-reinforced MTMS aerogel: (**A**) biomass growing into monolithic PP-reinforced MTMS aerogel, (**B**) elements of disintegrated MTMS aerogel attached to biomass.

*Rindera graeca* hairy roots unequivocally show affinity to MTMS-based aerogel. As shown in Figure 10, roots grown on MTMS-based aerogel constructs penetrated their pores or attached its fragments. Such a phenomenon was never observed in the case of PUF applied as biomaterial. Observed affinity might additionally prove the biocompatibility of MTMS aerogel. Literature data suggest that surface charge affects the adhesion of cells, e.g., plants [27], roots [28] or microorganisms [29]. The surface of MTMS-based aerogel is positively charged resulting from the presence of -CH_3_ groups. Such effects induced adhesion of cells to positively charged MTMS aerogel, in contrast to negatively charged polyurethane groups in PUF (Figure 1). Lipophilic nature of naphthoquinones might also play a significant role in their enhanced production observed for the culture system supported with constructs characterized by surfaces rich in hydrophobic/lipophilic organic groups, as MTMS aerogel [30,31]. Furthermore, the negative effects of feedback inhibition of metabolite biosynthesis can be limited by in situ extraction of the desired metabolite from the culture medium, what has been previously discussed [32] and reviewed [33].

The highest biomass growth and naphthoquinones production was noted for the culture system supplemented with MTMS aerogel applied in disintegrated form. However, it must be emphasized that the monolith constructs are much easier to handle. Elements of disintegrated aerogel require more careful handling at stage of sample harvesting and product extraction.

## 5. Conclusions

The feasibility and applicability of a novel bioengineering approach toward producing secondary plant metabolites in hairy roots in vitro cultures was introduced and discussed. MTMS aerogel was presented as a bifunctional biomaterial for simultaneous immobilization of *R. graeca* roots and induction of naphthoquinones productivity, as well as for improved in situ extraction of biosynthesized metabolites. Moreover, organosilica-based MTMS aerogel, applied in disintegrated or monolithic forms clearly exhibited biocompatibility resulting in increased biomass growth compared to values noted for culture systems supported with PUF, PP fibers and the reference culture of nonimmobilized hairy roots. High level of selective adsorption of lipophilic metabolites exhibited by MTMS aerogel allow to recognize them as a suitable material for the enhanced in situ extraction of extracellularly secreted naphthoquinones. However, further investigations on the biomass growth kinetics and detailed characterization of naphthoquinones production dynamics must be performed. Summarizing, application of MTMS aerogel in culture systems for hairy roots bioprocessing allows for more effective exploitation of unique biosynthetic abilities of plant biomass according to improved downstream processing of the desired metabolite.

## Figures and Tables

**Table 1:** Basic properties of PUF, MTMS aerogel, PP fibers and PP-reinforced MTMS aerogel applied in the current study.

Tested Material	Porosity (%)		Density (g cm^−3^)		Water/Air Contact Angle (°)	
	Value	*σ*_SD_	Value	*σ*_SD_	Value	*σ*_SD_
PUF	98.95	3.91 × 10^−2^	0.0127	4.69 × 10^−4^	86.56	5.02
MTMS aerogel	90.55	4.20 × 10^−3^	0.1370	5.43 × 10^−4^	156.0	1.86
PP fibers	97.27	6.71 × 10^−4^	0.0248	6.12 × 10^−4^	144.2	4.26
PP-reinforced MTMS aerogel	89.56	3.66 × 10^−3^	0.1258	4.41 × 10^−3^	155.8	1.76

**Table 2:** Values of *Y*_P/X_ and *μ* characterizing cultures of *R. graeca* hairy roots performed in systems supported with tested materials.

Culture System	*Y*_P/X_ (µg g_DW_^−1^)		*µ* × 10^3^ (h^−1^)	
	Value	*σ*_SD_	Value	*σ*_SD_
Nonimmobilized biomass (reference culture)	0.0	0.00	2.019	2.42 × 10^−2^
PUF	653.6	1.60 × 10^2^	1.134	3.02 × 10^−1^
MTMS aerogel (in disintegrated form)	636.2	7.98 × 10^1^	2.678	1.20 × 10^−1^
PP fibers (control culture)	0.0	0.00	1.790	8.10 × 10^−2^
PP-reinforced MTMS aerogel (in monolithic form)	46.04	6.71	2.169	1.88 × 10^−1^

**Table 3:** Previously published data on cultures of transgenic roots immobilized on various materials.

Plant Species	Bioproduct (Type of Metabolite)	Material Construct	*V*_L_ (dm^3^)	*Y*_P/X_ (µg g_DW_^−1^)	*µ* × 10^3^ (h^−1^)	References
*Arachis hypogaea*	Resveratrol (stilbene)	DIAION HP-20 polystyrene resin	0.05	11 976	n/a	[24]
*Azadirachta indica*	Azadirachtin (tetranortriterpenoid)	PP mesh	1	2100	1.68	[10]
		PUF	1	3100	1.69	
			1	6400	2.42	[11]
*Catharanthus roseus*	Ajmalicine (alkaloid)	PUF	3	1130	1.76	[12]
*Plumbago rosea*	Plumbagin (naphthoquinone)	PP disk	1	1500	3.57	[13]
*Rindera graeca*	Naphthoquinones	PUF	0.05	653	1.13	Current study
		MTMS aerogel	0.05	636	2.68	
*Salvia miltiorrhiza*	Diterpenoides fraction	X-5 polystyrene resin	0.05	550	n/a	[25]
		Amberlite XAD-4	0.05	550	n/a	
*Tagetes patula*	Thiophenes fraction	Amberlite XAD-7	0.05	294	1.80	[26]

## Data Availability

The data presented in this study are available on request from the corresponding author.
